# Maltodextrin Consumption Impairs the Intestinal Mucus Barrier and Accelerates Colitis Through Direct Actions on the Epithelium

**DOI:** 10.3389/fimmu.2022.841188

**Published:** 2022-03-14

**Authors:** Megan T. Zangara, András K. Ponti, Noah D. Miller, Morgan J. Engelhart, Philip P. Ahern, Naseer Sangwan, Christine McDonald

**Affiliations:** ^1^Department of Molecular Medicine, Cleveland Clinic Lerner College of Medicine, Case Western Reserve University, Cleveland, OH, United States; ^2^Department of Inflammation & Immunity, Lerner Research Institute, Cleveland Clinic, Cleveland, OH, United States; ^3^Department of Biology, John Carroll University, University Heights, OH, United States; ^4^Department of Cardiovascular & Metabolic Sciences, Lerner Research Institute, Cleveland Clinic, Cleveland, OH, United States; ^5^Microbiome Composition and Analytics Cores, Lerner Research Institute, Cleveland Clinic, Cleveland, OH, United States

**Keywords:** goblet cells, maltodextrin, processed food, inflammatory bowel disease, mucus, microbiome

## Abstract

Food additives are common components of processed foods consumed in a Western diet. In inflammatory bowel disease patients, some diets that exclude food additives improved clinical disease parameters, suggesting a link between food additives and disease pathogenesis. Food additives also enhanced disease severity in mouse colitis models through incompletely described mechanisms. This study examined the mechanisms by which the food additive maltodextrin (MDX) alters the development of colitis in a murine model. Interleukin-10 knockout (IL10KO) mice were fed diets supplemented with MDX or carboxymethyl cellulose (CMC) to determine their impact on colitis onset and severity; microbiome composition, function, and location; colonic immune cell infiltrates; and mucus layer integrity. Primary IL10KO colonic epithelial monolayers were used to dissect the impact of MDX directly on epithelial differentiation and mucus production. MDX or CMC consumption increased the incidence and severity of colitis, as well as decreased microbiome diversity, altered microbial composition, and decreased fecal acetic acid levels. The number of mucus producing cells were decreased in food additive fed mice and resulted in increased microbial proximity to the intestinal epithelium. Additionally, MDX supplementation resulted in crypt hyperplasia and expansion of the HopX+ injury renewal stem cell niche. In primary intestinal epithelial-derived monolayers devoid of microbes and immune cells, MDX exposure decreased goblet cell number and mucus production in association with downregulated expression of the transcription factor *Klf4*, a marker of terminally differentiated goblet cells. These results suggest MDX disrupts the balance of epithelial cell differentiation and proliferation to contribute to disease pathogenesis through direct and indirect actions on the intestinal epithelial barrier.

## Introduction

Inflammatory bowel disease (IBD) was first described in Western populations; however, IBD prevalence worldwide is on the rise in parallel with societal industrialization and consumption of a “Western-style” diet (1–3). A Western-style diet is comprised of high consumption of pre-packaged foods, processed meat, red meat, high-fat dairy products, refined grains, sweets, and high-sugar drinks, with low amounts of whole grains, fruits, vegetables, fish, nuts, and seeds. Although correlations exist between the introduction of processed foods and a rise in IBD prevalence, the specific components of processed foods and the mechanisms by which they drive disease pathogenesis are not fully understood (4).

Food additives are a broad class of compounds used to preserve taste, appearance, and texture, as well as add bulk to processed food products (5). Despite a Food and Drug Administration classification of Generally Recognized As Safe (GRAS), several food additives have been implicated in promoting or enhancing gastrointestinal inflammation in humans. Carrageenan, a food additive used as an emulsifying and thickening agent, has been associated with accelerated disease relapse in ulcerative colitis patients (6). The emulsifiers polysorbate-80 and carboxymethyl cellulose (CMC) were shown to increase pro-inflammatory potential of the human intestinal microbiota by upregulating lipopolysaccharide and flagellin levels using an *in vitro* human microbiota culture system (7). Although data on the direct effect of food additives on disease in humans is limited, small studies on the effects of elimination diets have provided additional evidence for their potential role as disease agonists. Diets such as the specific carbohydrate diet (SCD), low fermentable oligo-, di-, and mono-saccharides and polyols (FODMAP) diet, and Crohn’s Disease Exclusion Diet, which eliminate many carbohydrates and all processed foods, have shown modest success in relieving functional gastrointestinal symptoms and decreasing markers of inflammation in IBD patients (8–12).

Using pre-clinical rodent models, food additives have been shown to have a number of deleterious effects on intestinal homeostasis through action on intestinal microbes (7, 13–17). Maltodextrin (MDX), a polysaccharide derived from the chemical and enzymatic processing of natural starches, increased pathogenic phenotypes of Crohn’s disease-associated adherent invasive *Escherichia coli* strains, and disrupted host defenses against the enteric pathogen *Salmonella enterica* serovar Typhimurium (16, 17). While MDX consumption by wild-type mice has not been shown to initiate spontaneous intestinal inflammation on its own, MDX exacerbated inflammation in an acute dextran sodium sulfate (DSS) model of intestinal injury (14). Similarly, the emulsifiers polysorbate-80 and CMC have been closely investigated using spontaneous colitis models that allow for the investigation of dietary influence in a model that combines genetic risk factors and microbial-dependent inflammation. Both emulsifiers were shown to accelerate colitis incidence through alterations to microbiome composition, bacterial function, and increased proximity to the intestinal epithelium in colitis-prone mice (13). These data suggests that food additives are not inert filler compounds and warrant closer investigation in inflammatory disease models.

The intestinal mucus layer is an important protective barrier that separates the intestinal epithelium from the commensal microbes of the host microbiome. In IBD patients, the mucus layer integrity is decreased, allowing for microbial interaction with the epithelium and leading to an inflammatory response (18). Previous studies in mice have described detrimental effects of food additives on intestinal mucus layer integrity, but the mechanism by which this happens still remains unknown (13, 14). Additionally, it is not known whether the food additives directly disrupt the mucus layer through their surfactant properties, upregulate the mucolytic activity of the intestinal microbiome, or suppress mucus production from the intestinal epithelium to facilitate this phenotype.

In this study, we investigated mechanisms of food additive-induced impairment of mucus barrier integrity in the microbe-dependent interleukin-10 deficient (IL10KO) mouse colitis model. We determined that two common food additives, MDX and CMC, accelerate colitis onset and severity in the IL10KO mouse model. Both food additives altered microbiome localization, composition, and function, as well as greatly decreased host mucus layer integrity. Using differentiated, mucus-producing primary intestinal epithelial cell-derived monolayers, we determined that decreased mucus production was caused by direct action of MDX on intestinal epithelial cells, while additional signals were required to drive intestinal crypt hyperplasia characterized by epithelial proliferation and an expanded HopX+ injury renewal stem cell niche. These findings demonstrate that MDX disrupts the balance of epithelial cell differentiation and proliferation to contribute to disease pathogenesis. Our results identify an additional, epithelium-specific mechanism by which food additives alter intestinal homeostasis that combines with microbiome dysbiosis, and immune cell dysfunction to accelerate colitis development.

## Results

### Food Additive Consumption Accelerates Colitis Onset in Interleukin-10 Deficient Mouse Model

**Figure 1:**
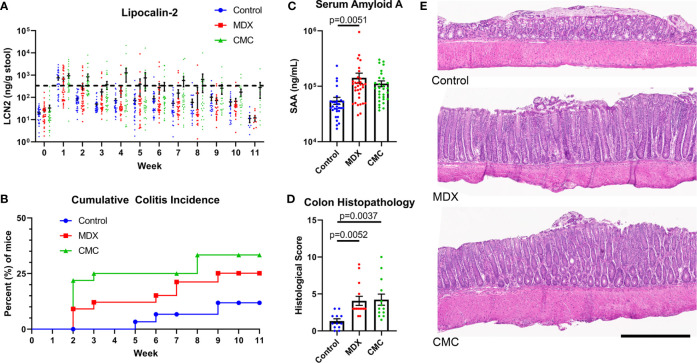
MDX and CMC increase colitis incidence in genetically susceptible hosts. IL10KO mice were conditioned with fecal material from NOD2KO mice for up to 11 weeks and concurrently fed a control chow diet or a food additive supplemented diet containing either 1% w/w MDX or CMC. **(A)** Fecal lipocalin-2 (LCN2) levels were assessed from fresh stool samples collected weekly to monitor intestinal inflammation levels. A LCN2 value of 500ng/g of stool (dotted line) post week 2 was used as a benchmark to determine colitis status. Samples from 5 independent experiments, with 3-12 mice per diet per experiment. **(B)** Cumulative incidence of colitis over time by diet group, represented as percent of mice that developed colitis. A LCN2 value of 500ng/g of stool or greater after week 2 was used to determine colitis onset. Once a mouse reached the threshold, an incident was counted. Any mice who did not reach the threshold at any point throughout the experiment were censored at the endpoint. Mean ± SEM, statistical significance for trend was determined by logrank test. **(C)** Serum amyloid A levels were assessed in the plasma isolated from whole blood collected at endpoint. Samples from 5 independent experiments, with 3-12 mice per diet per experiment. Mean ± SEM, statistical significance was determined by one-way ANOVA with Dunnett’s multiple comparisons test. **(D)** Histological score of H&E colon sections. Score is a cumulative total of the 4 assessed parameters: epithelial layer integrity, immune cell infiltration, submucosal swelling, and muscularis hyperplasia. Samples from 4 independent experiments, with 2-5 mice per diet per experiment were scored. Mean ± SEM, statistical significance was determined by one-way ANOVA with Dunnett’s multiple comparisons test. **(E)** Representative H&E stained colon tissue sections. Scale bar is 500μm.

Using IL10KO mice, we investigated whether MDX consumption accelerated spontaneous colitis onset similar to reports of another food additive, CMC (13). Due to the high microbial dependence of the model (19), IL10KO mice were conditioned with fecal material from NOD2KO mice to introduce a pro-inflammatory microbiota and normalize disease onset between cages. Mice were fed a grain-based rodent chow supplemented with 1% w/w MDX or 1% w/w CMC for up to 11 weeks. Fecal lipocalin-2 (LCN2) levels were measured to monitor intestinal inflammation levels and a threshold of 500ng/g of stool was used to define colitis onset as previously described (13). In IL10KO mice, MDX supplementation caused an increase in colitis incidence and earlier disease onset as assessed by fecal LCN2 levels (**Figure 1A**). Both food additive-supplemented diets increased disease incidence: 11% of mice fed an unsupplemented control diet developed colitis while 25% of mice consuming a MDX-supplemented diet and 33% consuming a CMC-supplemented diet developed colitis (p=0.0399; **Figure 1B**). Disease onset was accelerated from 5 weeks in the control group to 2 weeks in both food additive diet groups. Food additive-fed IL10KO mice had elevated serum amyloid A (SAA) levels indicating increased systemic inflammation in addition to enhanced colon histopathology (**Figures 1C, D**). The increased histopathology showed hallmarks of chronic inflammation and the colitis score was mainly driven by exacerbated crypt hyperplasia and local elevation of immune cell infiltrates (**Figure 1E** and **Supplementary Figure 1A**). No change in rates of weight gain, differences in diet consumption, or elevation in an observational disease activity index were detected (**Supplementary Figures 1B–D**). These findings demonstrate that MDX or CMC consumption increased colitis incidence, severity, and accelerated time to disease onset in colitis-prone IL10KO mice.

### Intestinal Immune Cell Populations Are Not Impacted by Food Additive Consumption

**Figure 2:**
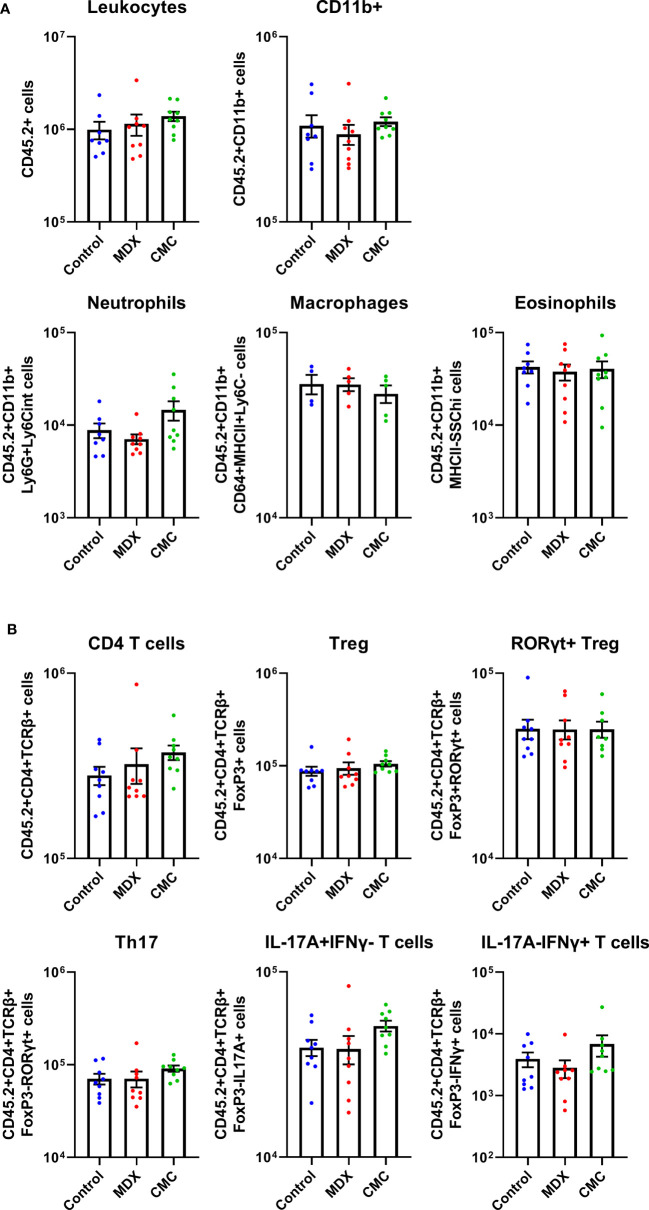
Acceleration of colitis onset by food additives is not driven by the immune compartment. Flow cytometry was used to assess innate cell **(A)** and T cell **(B)** counts in the lamina propria cell population found in the proximal colon tissue of IL10KO mice. Samples from 2 independent experiments with 4-5 mice per diet per experiment. Mean ± SEM, statistical significance was determined by one-way ANOVA with Dunnett’s multiple comparisons test.

In the IL10KO colitis model, loss of IL-10 signaling removes inhibition of pro-inflammatory signaling, resulting in an influx of immune cells into the colon. A limited survey of innate and T cell populations isolated from the proximal colonic lamina propria by flow cytometry revealed that neither food additive grossly altered immune cell infiltrates as compared to the control-fed group (**Figure 2**). Total numbers of CD11b+Ly6G+Ly6Cint cells (neutrophils), CD11b+CD64+MHCII+Ly6C- cells (macrophages), and CD11b+MHCII-Ly6G-SSChi cells (eosinophils) were unaffected by diet (**Figure 2A**). The levels of Treg cells and pro-inflammatory Th17, IL-17A-producing, and IFNγ-producing T cells were also equivalent across diet groups (**Figure 2B**). This indicates that dietary additives do not produce large shifts in the surveyed pool of tissue-infiltrating immune cells, suggesting that alterations to immune cell infiltrates may not be the main driver of accelerated disease onset in response to food additive consumption.

### Food Additive-Driven Alterations to the Microbiome Correlate With Lower Short-Chain Fatty Acid Levels

**Figure 3:**
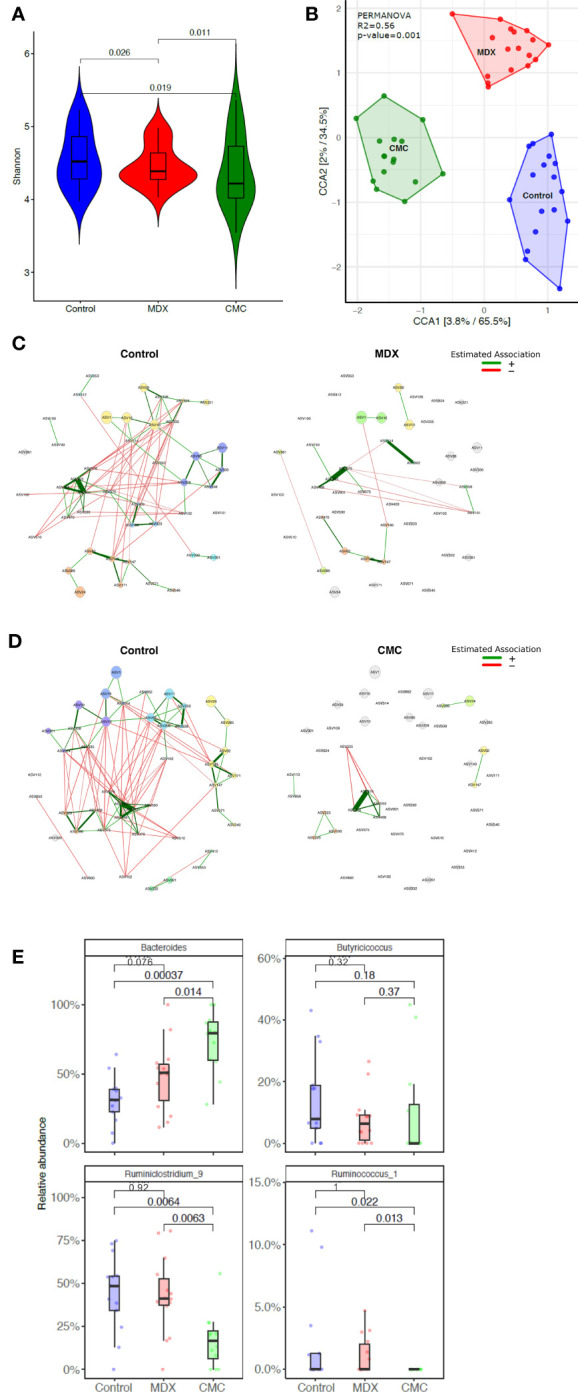
Food additives alter microbiome composition. Cecal contents from food additive fed IL10KO mice were sequenced in the V4 region of bacterial 16s rRNA. Samples from 4 independent experiments with 3-7 samples per diet per experiment. **(A)** Alpha diversity of cecal contents samples was assessed using the Shannon Index. Statistical significance was determined by ANOVA. **(B)** Amplicon sequence variants (ASVs) were used to assess beta diversity, and is represented by canonical correspondence analysis (CCA) plot. Statistical significance was determined using PERMANOVA. **(C, D)** Comparison of bacterial associations in the cecal samples between the different diets (i.e. CMC and Control). The SPRING method was used as the association measure. The estimated partial correlations are transformed into dissimilarities *via* the “signed” distance metric and the corresponding similarities are used as edge weights. Eigenvector centrality was used for defining hubs and scaling node sizes. Node colors represent clusters, which are determined using greedy modularity optimization. Clusters have the same color in both networks if they share at least two taxa. Green edges correspond to positive estimated associations and red edges to negative ones. The layout computed for the WD network is used in both networks. Nodes that are unconnected in both groups are removed. Taxa names are abbreviated as numbered ASVs (**Supplementary Table 1** for full names). **(E)** Differential abundance of select genera significantly alter by food additive supplementation. Abundance is represented as a percent of total microbes surveyed. Statistical significant was determined using the Wilcoxon ranked-sign test.

Commensal microbes have direct exposure to food products in the intestine, and diet composition impacts the composition and functions of the microbiome. Changes to the diet, such as a shift towards eating a Western-style diet, have been implicated to be pathogenic, either driving disease or contributing to enhancing inflammation. Both MDX and CMC supplementation caused a decrease in alpha diversity as compared to the control diet, indicating a decrease in the complexity of the microbiome (**Figure 3A**). Comparison of the relative abundance of bacterial phyla did not demonstrate any high-level changes between the diet groups (**Supplementary Figure 2A**); however, canonical correspondence analysis determined that food additive diets caused distinct strain level shifts, resulting in three distinct, diet-driven community compositions (**Figure 3B**). A network analysis of amplicon sequence variants (ASV) using the SPRING method revealed that both food additives caused drastic alterations to interactions between several microbial taxa, resulting in loss of connections between taxa found in the control group, as well as the establishment of new connections between different taxa in the food additive fed samples (**Figures 3C, D**).

Among the various microbes whose abundance was differentially altered by either of the food additives, 8 genera were altered by at least one food additive compared to the control diet, with a trending change in abundance in 12 additional genera (**Supplementary Figure 2B**). Of particular interest were decreases in *Ruminoclostridium* and *Ruminococcus*, and an increase in *Bacteroides* abundance, as these findings parallel reports in IBD patient microbiomes of altered abundance in these genera (20) (**Figure 3E**).

**Figure 4:**
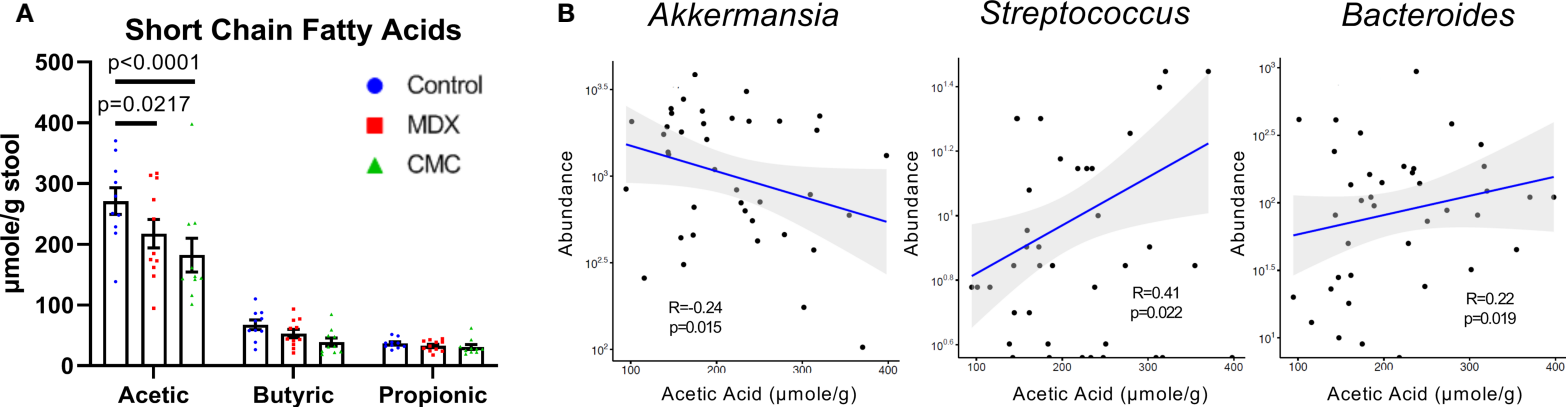
Food additives alter microbial short-chain fatty acid production. **(A)** Short chain fatty acid levels were assessed in cecal contents by GC-MS/MS. Samples from 3 independent experiments, with 2-6 samples per diet per experiment. Mean ± SEM, statistical significance was determined by two-way ANOVA with Dunnett’s multiple comparisons test. **(B)** Relative microbial abundance correlated with acetic acid levels from corresponding cecal contents samples. Samples from 3 independent experiments, with 2-6 samples per diet per experiment. Statistical significance was determined *via* linear regression analysis.

Short-chain fatty acids (SCFA), such as acetic, butyric, and propionic acid, are fermentation byproducts of the gut microbiome which serve many biological roles such as modulating inflammation and immunity, and in particular butyric acid is an important energy source for colonocytes. Decreased SCFA levels have been implicated in the inflammation of many diseases, including IBD (21). Assessment of SCFA levels in cecal contents by GC-MS/MS revealed that both MDX and CMC consumption caused a decrease in acetic acid levels, but not butyric or propionic acid (**Figure 4A**). The altered acetic acid levels correlated with the abundance of several microbes; lower levels of fecal acetic acid correlated with higher abundance of *Akkermansia*, and lower abundance of *Streptococcus* and *Bacteroides* (**Figure 4B**). Overall, this data demonstrates that both MDX and CMC alter the microbiome community structure as well as its function, which may contribute to colitis acceleration.

### Food Additives Decrease Goblet Cell Numbers and Increase Microbial Proximity to the Intestinal Epithelium

**Figure 5:**
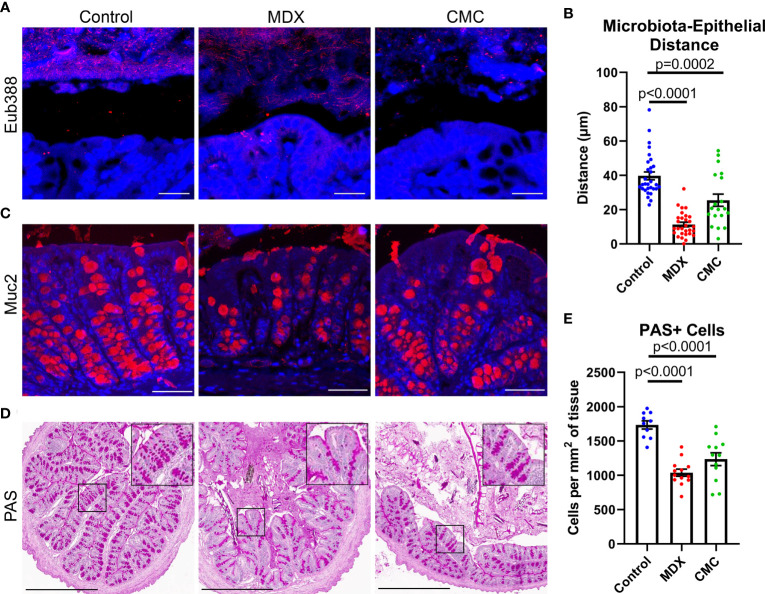
Food additives alter bacterial localization and mucus production *in vivo*. **(A)** Representative confocal microscopy images taken at 63x magnification of Carnoy’s-fixed proximal colon cross-sections visualizing the location of commensal microbes by FISH (Eub388, red). Scale bar is 25μm. **(B)** Assessment of distance between commensal microbes and the intestinal epithelium. Samples from 3 independent experiments, with 2 mice per experiment per diet. Mean ± SEM, statistical significance was determined by one-way ANOVA with Dunnett’s multiple comparisons test. **(C)** Representative confocal microscopy images taken at 40x magnification immunofluorescent Muc2 (red) and DAPI stained Carnoy’s-fixed proximal colon cross-sections. Scale bar is 50μm. **(D)** Representative PAS-stained Carnoy’s-fixed proximal colon cross-sections. Inset shows magnified view of PAS stained cells. Scale bar is 500μm. **(E)** Quantification of PAS-positive cells per proximal colon cross-section tissue area. Samples from 3 independent experiments, with 3-5 mice per experiment were scored. Mean ± SEM, statistical significance was determined by one-way ANOVA with Dunnett’s multiple comparisons test.

Alterations to microbial composition in the intestine can often be concomitant with increased microbial proximity to the epithelium as microbial populations expand and create new niches. Fluorescent *in situ* hybridization (FISH) of Carnoy’s-fixed intestinal tissue revealed that the distance between the commensal microbes and the epithelium was significantly decreased in MDX- and CMC-fed mice (**Figures 5A, B**). This data suggests an alteration in mucus layer integrity, allowing for the bacteria of the microbiome to reside in closer proximity to the epithelium.

The mucus layer is vital to maintaining intestinal homeostasis by creating a physical and chemical barrier between the epithelium and the commensal microbes that reside in the intestinal lumen. Alterations to mucus production in goblet cells results in compromised mucus layer integrity, which can lead to increased proximity of microbes to the epithelial cell layer. Both food additives caused a visual reduction in mucin 2 (Muc2) epithelial staining (**Figure 5C**). Quantification of Periodic Acid Schiff (PAS) positive cell numbers revealed a decrease in mucus containing cells (**Figures 5D, E**). The combination of increased bacterial encroachment on the intestinal epithelium and reduction in Muc2 protein and mucin levels suggests that both food additives are decreasing mucus layer integrity, through a reduction in goblet cell number.

### Maltodextrin Suppresses Mucus Production by Acting Directly on Intestinal Epithelial Cells

**Figure 6:**
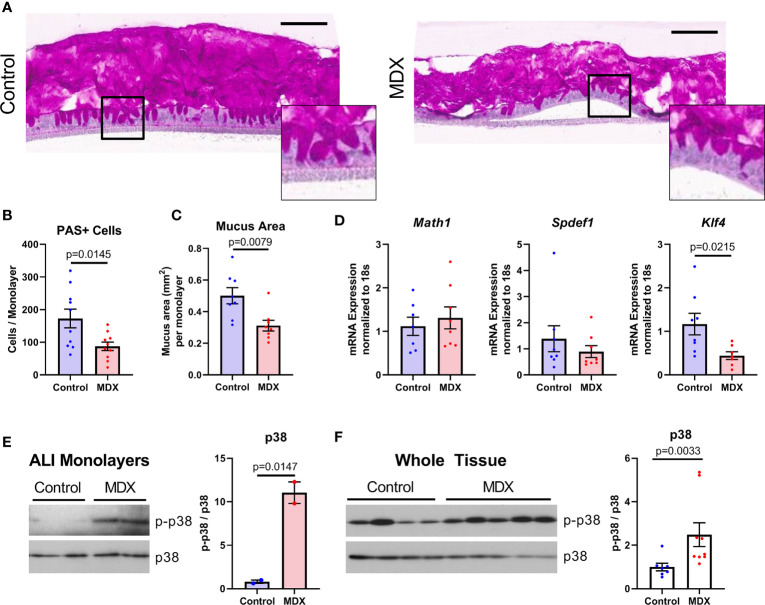
Mucus depletion by MDX is by direct action on intestinal epithelial cells through downregulation of differentiation factor *Klf4*. Primary intestinal epithelial-derived monolayers from IL10KO distal colon tissue were cultured in air-liquid interface conditions. Monolayers were supplemented for 72 hours with MDX. **(A)** Representative PAS-stained sections of Carnoy’s-fixed monolayers. Inset shows magnified view of PAS positive staining cells. Scale bar is 100μm. **(B, C)** Quantification of PAS-positive cells per monolayer and total mucus area per monolayer. Samples from 3 independent experiments, 2-6 monolayers per experiment were assessed. Mean ± SEM, statistical significance was determined by two-tailed unpaired t-test. **(D)** Relative expression levels of goblet cell markers in ALI epithelial monolayers as assessed by qRT-PCR. All expression levels normalized to 18s ribosomal transcript levels. Samples from 3 independent experiment, with 2-3 monolayers per experiment. Mean ± SEM, statistical significance was determined by two-tailed unpaired t-test. **(E)** Immunoblot quantification of phosphorylated p38 levels in protein lysates from ALI cultures (n=2 monolayers). Phosphorylated p38 (p-p38) levels were normalized to total p38 levels. Mean ± SEM, statistical significance was determined by two-tailed unpaired t-test. **(F)** Immunoblot quantification of phosphorylated p38 levels in proximal colon tissue lysates from IL10KO mice. Samples from 2 independent experiments, with 3-5 samples per diet per experiment. Mean ± SEM, statistical significance was determined by two-tailed Mann-Whitney test.

Mucus layer production and integrity is influenced by microbial and host-derived signals, both of which can be altered with inflammation. The effects of the emulsifier CMC on mucus layer integrity has been demonstrated to be mediated through changes in the microbiome rather than a direct effect of CMC on the epithelium (13). Strain level alterations to the microbiome composition differed between MDX- and CMC-fed mice, therefore we sought to investigate the requirement of microbes in the reduction of mucus observed with MDX supplementation. Primary IL10KO intestinal epithelial-derived monolayers, when cultured under air-liquid interface (ALI) conditions, develop fully differentiated goblet cells and secrete a robust mucus layer (**Figure 6A**). Exchanging half of the glucose in the lower-chamber media for an equivalent concentration of MDX for 72 hours resulted in a significant reduction of both mucus-secreting cell numbers and total area of secreted mucus, as assessed by quantification of PAS staining (**Figures 6B, C**).

Goblet cells constitutively produce and steadily secrete mucus, beginning soon after differentiation from the stem cell niche found in the base of intestinal crypts (22). The goblet cell precursors mature as they transit away from the crypt base and reach full differentiation near the luminal epithelial surface (22). Reduced mucus production was associated with a decrease only in the terminally differentiated goblet cell marker *Klf4*, with no observed reduction in secretory cell lineage marker *Math1* or immature goblet cell marker *Spdef1* transcript levels (**Figure 6D**). These results indicate that MDX has a direct impact on goblet cell differentiation in the absence of microbial or immune signals.

A prior study indicated that MDX consumption induced endoplasmic reticulum stress and activation of the mitogen activated protein kinase p38 (14). Immunoblot analysis of protein lysates from both ALI monolayers and intestinal tissue from IL10KO mice demonstrated MDX-induced enhanced phosphorylation of p38 (**Figures 6E, F**). However, no upregulation of an IRE1-mediated ER stress response pathway was observed (**Supplementary Figure 3**). This data suggests that MDX acts directly on intestinal epithelial cells to alter mucus production by activating p38 kinase activity and impairing terminal differentiation of goblet cells in the absence of microbes or immune cells.

### Epithelial Proliferation and Microbe Sensors Are Increased in MDX-fed IL10KO Mice

**Figure 7:**
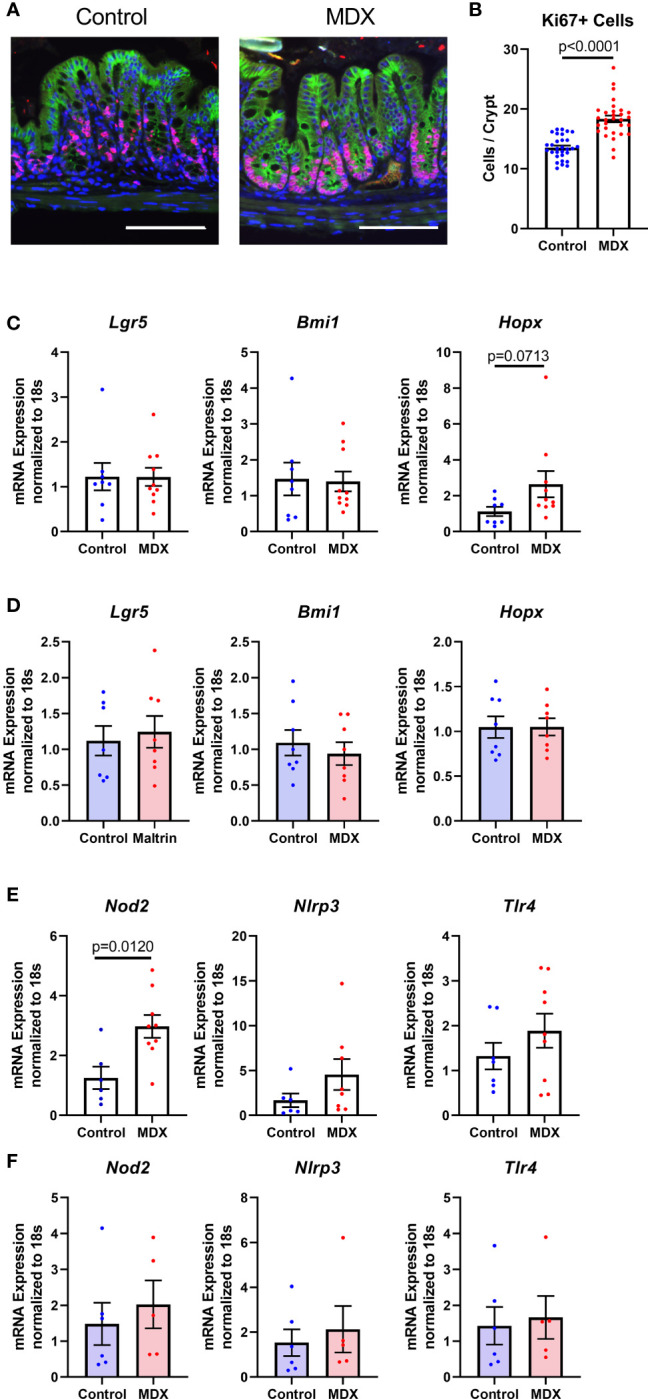
MDX increases epithelial proliferation and injury renewal cell population *in vivo*. **(A)** Representative immunofluorescent confocal micrographs of Ki67 (red), E-cadherin (green), and DAPI stained proximal colon cross-sections. Scale bar is 100μm. **(B)** Quantification of Ki67-positive cells present per intestinal crypt unit. Samples from 3 independent experiments, with 2 mice per diet per experiment were assessed. Mean ± SEM, statistical significance was determined by two-tailed Mann-Whitney test. **(C)** Relative transcript levels of stem cell markers in isolated primary intestinal epithelial cells assessed by qRT-PCR. All expression levels normalized to 18S ribosomal transcript levels. Samples from 7-10 mice per diet. Mean ± SEM, statistical significance was determined by two-tailed Mann-Whitney test. **(D)** Relative transcript levels of stem cell markers in ALI monolayers assessed by qRT-PCR. All expression levels normalized to 18S ribosomal transcript levels. Samples from 3 independent experiments, with 2-3 monolayers per experiment. Mean ± SEM, statistical significance was determined by two-tailed unpaired t-test. **(E)** Relative expression levels of pattern recognition receptors in whole colon tissue samples as assessed by qRT-PCR. All expression levels normalized to 18S ribosomal transcript levels. Samples from 2 independent experiments, with 3-5 sampled per diet per experiment. Mean ± SEM, statistical significance was determined by two-tailed Mann-Whitney test. **(F)** Relative expression levels of pattern recognition receptors in ALI monolayers as assessed by qRT-PCR. All expression levels normalized to 18S ribosomal transcript levels. Samples from 3 independent experiments, with 2-3 monolayers per experiment. Mean ± SEM, statistical significance was determined by two-tailed unpaired t-test.

The intestinal epithelium is a site of steady proliferation and regeneration, with complete turnover of cells every three to four days under homeostatic conditions. Rapid proliferation is essential in response to epithelial damage, such as increased contact with resident microbiota. Crypt hyperplasia was observed in the colons of MDX-fed IL10KO mice (**Figure 1E**), with a significant increase in the number of proliferative, Ki67-positive cells at the crypt base where stem cell populations are enriched (**Figures 7A, B**). To determine if these Ki67+ cells were an expansion of a specific stem cell population, transcript levels of the three major stem cell type markers (*Lgr5, Bmi1*, and *Hopx*) were assessed in isolated intestinal epithelial cells from IL10KO mice. The transcript levels of *Hopx*, which is described as a marker of injury renewal stem cells, were modestly elevated in isolated intestinal epithelial cells, but this increase did not reach statistical significance (p=0.0713; **Figure 7C**). This increase in *Hopx* expression was not observed in MDX-supplemented ALI monolayers (**Figure 7D**), suggesting that additional factors are required to drive this injury response.

**Figure 8:**
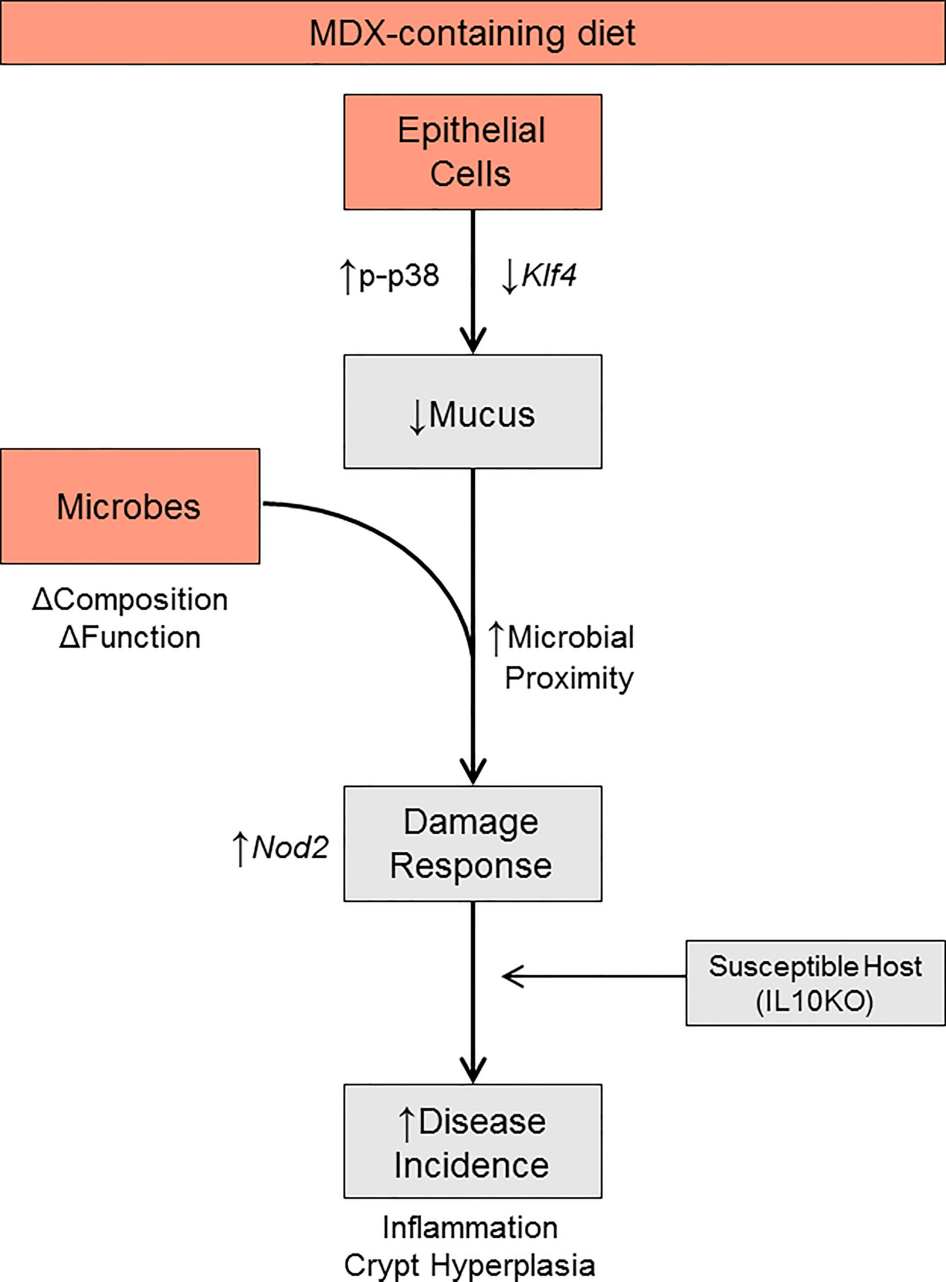
Proposed model. MDX directly acts on intestinal epithelial cells to increase p38 MAPK stress signaling, reduce *Klf4* transcript levels, and decrease mucus production. Concurrently, MDX alters the composition and function of commensal microbes. Combined with the reduction in mucus, microbial proximity to the epithelium is increased and elicits a damage response characterized by crypt hyperplasia through upregulated NOD2 signaling. In a genetically susceptible host, these direct and indirect actions of MDX results in accelerated disease incidence.

Due to the observation that MDX-supplementation caused an increase in proximity of the commensal microbes to the intestinal epithelium, the expression of pattern recognition receptors was analyzed to determine whether microbial signaling could contribute to the epithelial hyperproliferation in MDX fed mice. Expression of *Nod2*, an intracellular receptor that recognizes both Gram-positive and Gram-negative bacteria, was significantly increased in intestinal tissue from MDX fed mice, while transcripts of other bacterial sensors, *Nlrp3* and *Tlr4* were not (**Figure 7E**). When examined in the ALI monolayer culture system, expression of these bacterial sensors was unaffected by MDX supplementation, suggesting a role for additional signals from the intestinal environment in the hyperproliferative response to MDX consumption (**Figure 7F**). Taken together, this data suggests that MDX has direct effects on the intestinal epithelium that result in an impaired mucus barrier, as well as indirect effects on the proliferation of the stem cell niche that may be mediated by other factors such as microbes in closer proximity to the epithelium or stimulation of immune cells in proximity to the epithelium (**Figure 8**).

## Discussion

Inflammatory bowel disease is a complex and multi-factorial disease. Growing epidemiological and laboratory research points towards components of an increasingly prevalent Western-style diet as a factor in disease onset and progression (3, 23–25). Dietary composition is an easily modifiable factor, and diet-based therapies have been proven effective in treating other chronic diseases, such as type II diabetes mellitus (26). While there is rising interest in the IBD patient community for diet modification protocols to treat disease symptoms, these diets are often broadly restrictive and are challenging to implement and maintain. Therefore, a comprehensive understanding of how diet influences disease pathogenesis would allow for the development of more specific dietary interventions that remove components of the Western diet that aggravate disease symptoms.

In this study, we investigated two common food additives, MDX and CMC, using a colitis-prone mouse model driven by both host genetics and microbes, allowing us to investigate the integration of several risk factors on chronic disease activity. Both MDX and CMC accelerated the onset and severity of intestinal and systemic inflammation, altered the microbiome profile and function, and decreased intestinal mucus production. This data reproduces prior findings implicating emulsifier-induced microbial dysbiosis and bacterial encroachment of the mucus barrier in acceleration of disease in two different genetically-driven spontaneous colitis models, while extending these findings to identify differences in mechanisms of food additive action. While our group has previously shown that consumption of a high concentration of MDX (5% MDX for 2 weeks) alters commensal microbe localization in the intestine, we determined in this study that even a concentration as low as 1% can induce similar changes (16). Unlike previous reports on the emulsifiers CMC and polysorbate-80 which have been shown to induce large high level shifts of whole bacterial phyla (13), MDX caused strain-level alterations to microbial abundance, leading to altered microbial network interactions. This matches previous observations that MDX did not cause alterations to mucosa-associated microbiota at the phyla and class level (14), suggesting an additional or alternate mechanism that contributes to disease acceleration. We identified an epithelial-specific response to MDX that leads to decreased mucosal barrier integrity. Combined with subtle alterations to the microbiome composition and increased proximity of the microbiome to the epithelium, MDX consumption increased colitis incidence and disease severity in genetically-prone mice. Our current work reiterates that food additives are not inert compounds and can combine with other risk factors to increase colitis susceptibility (**Figure 8**). Given that MDX and CMC both accelerate colitis through different mechanisms, future studies that examine the potential for combinatorial or synergistic action of food additives on disease would further refine our understanding of how processed foods that often contain multiple food additives impact disease risk in susceptible individuals or could be potential triggers for individuals with IBD. This type of study could also elucidate critical pathways contributing to disease pathogenesis and highlight key areas of intervention.

Decreased mucus production is a hallmark of ulcerative colitis, resulting in compromised mucus layer integrity and increased microbial-epithelial contact (18). Studies of CMC and polysorbate-80 linked decreased mucus layer integrity to an increased abundance of mucolytic bacteria, which we did not observe in our study (13). Using an *in vitro* culture system of primary intestinal epithelial-derived monolayers that lack stromal and immune cells, as well as microbes, we determined that impaired mucus barrier integrity and decreased goblet cell numbers are a result of MDX acting directly on intestinal epithelial cells. One proposed mechanism for MDX-stimulated mucus depletion is activation of an ER stress response, as a study investigating the effect of MDX on disease severity in an acute DSS colitis model linked mucus depletion to increased p38 activation and enhanced ER stress mediated by IRE1β (14). Although we observed increased p38 phosphorylation in both intestinal tissue from MDX fed mice and ALI monolayers cultured in MDX containing media, we did not observe alterations in IRE1α/β signaling responses in IL10KO mice that consumed a MDX-enriched diet (**Supplementary Figure 3**). Therefore, the mechanism by which MDX directly induces a reduction in *Klf4* levels in epithelial cells is still under investigation.

*In vivo*, we observed increased epithelial proliferation, as well as a potential shift of quiescent HopX+ injury renewal stem cells to a more proliferative state, suggesting an epithelial damage response. Although it has been demonstrated that KLF4 acts to inhibit cellular proliferation and promote differentiation in the intestinal epithelium (27), our *in vitro* data indicates that reduction of *Klf4* levels in epithelial cells by MDX exposure was not sufficient to drive epithelial hyperplasia (**Figure 7**). However, this may be due to limitations with the *in vitro* model. The epithelial monolayers could only be exposed to the food additive from the basal rather than the apical surface that would better model the dietary route of exposure; replacement of media to the apical surface of these ALI monolayers results in rapid de-differentiation and induction of an injury response (28). Proliferation in these confluent monolayers of primary epithelial cells may also be limited due to contact inhibition of these non-transformed cells. Additionally, while the isolated epithelial cell culture system grants the ability to study epithelial-specific alterations to intestinal barrier physiology, it also lacks microbial cues and responses from other cell types found in intestinal tissue that may be important in shaping the response of the epithelium directly or indirectly to MDX.

The upregulation of *Nod2* levels in intestinal tissue from mice fed MDX, but not in MDX-supplemented ALI monolayers, suggests that additional immune or microbial signals may be required to induce a damage response that results in MDX-stimulated epithelial hyperproliferation. We postulate that epithelial exposure to microbial products may be facilitated in MDX fed mice through the increased proximity of the microbiota to the epithelium resulting from MDX mucus barrier impairment. Alternately, the selective upregulation of *Nod2* transcripts in intestinal tissue from MDX fed mice could reflect an expansion of the stem cell niche, rather than increased microbial sensing, as NOD2 is highly expressed in intestinal stem cells and serves a protective role against reactive oxygen species induced by ionizing radiation or mitochondrial stress (29). Also of consideration is the method by which the epithelium is exposed to MDX in each system, as this may impact cellular responses as well. The apical surface of the intestinal epithelium is exposed to dietary components *in vivo*, whereas in the *in vitro* ALI cultures MDX was added to the basal media.

*Klf4* is not only required for terminal differentiation of goblet cells, but also has a broader function as a negative regulator of WNT signaling. In mice with an intestinal epithelium-specific knockout of *Klf4*, loss of expression not only resulted in inhibited goblet cell differentiation, but also increased activation of WNT signaling components and downregulation of differentiation regulators (30). *Klf4* has also been shown to function as a tumor suppressor, with heterozygous loss of function enhancing tumor progression in *APC^min^* mice (31). Colitis-associated colorectal cancer (CAC) is a significant risk for IBD patients, and while our model did not result in spontaneous tumor formation after 11 weeks of diet supplementation, the combination of intestinal inflammation and epithelial hyper-proliferation induced by MDX may increase the risk of tumor formation when combined with a second hit.

In summary, our findings reinforce the growing body of knowledge that food additives are not inert substances, and when combined with a genetic risk factor can accelerate the onset of chronic colitis. This study has identified an epithelial-specific mechanism by which the food additive MDX alters mucus production and increases colitis susceptibility in a genetically-prone model. Diet therapy is gaining interest for treating IBD symptoms by both adult and adolescent IBD patients (27). In order to effectively design therapeutic diets, it is important to understand how different dietary components affect mucosal health, so that those with deleterious effects can be targeted and removed. Future studies investigating the effects of withdrawing MDX from the diet on goblet cell numbers, mucus barrier, and colonic hyperplasia will be important in determining if the damage is reversible, or if supplements to alleviate cell stress are required. Food additives such as MDX may also contribute to CAC risk due to their influence on *Klf4* expression and epithelial proliferation. In vulnerable populations, this may be a driving component of the rise in IBD incidence and co-morbidities and is an ideal target for diet-based therapies.

## Materials and Methods

### Animals

The experimental protocol was approved by the Cleveland Clinic Institutional Animal Care and Use Committee. NOD2KO (B6.129S1-Nod2^tm1Flv^/J, The Jackson Laboratory, stock number 005763) and IL10KO (B6.129P2-Il10^tm1Cgn^/J, The Jackson Laboratory, stock number 002251) mice were bred in specific pathogen-free housing. For each experiment, IL10KO mice from multiple litters were equally divided into each diet group and housed in cages containing between 2 and 5 mice. The results of multiple, independent experiments were pooled to result in the analysis of 16-17 mice per diet group as a means to reduce the impact of maternal- and cage-dependent microbiota variations on the study outcomes. Male and female 3 to 5 week old mice were fed an irradiated 2018 Teklad Global 18% Protein Rodent Diet (Envigo) supplemented with 1% w/w maltodextrin (Maltrin, dextrose equivalence of 10.7) or carboxymethyl cellulose (Sigma Aldrich, 419311-100G), or a re-pelleted control and provided non-acidified water for up to 11 weeks. Once per day for three days prior to initiation of experimental diet, IL10KO mice were gavaged with 100uL of a fecal homogenate slurry made from NOD2KO stool to induce a more pro-inflammatory microbiota as found in IBD and normalize the initial microbiota between cages. Briefly, fresh stool was collected from male NOD2KO mice, and suspended at a concentration of 100mg/mL in sterile PBS. Stool was vortexed to homogenize pellets, and the supernatant removed from the non-soluble particulates. The supernatant was then mixed in a 1:1 ratio with sterile 50% glycerol, aliquoted, and stored at -80°C prior to use. Food consumption and animal weight were monitored weekly. At time of weighing, fresh stool was collected, animals were gavaged with 100uL of fecal homogenate slurry, and clean cages were conditioned with 50 grams of soiled bedding from a NOD2KO cage. At completion of the experiment, blood was collected by cardiac puncture into microtainer EDTA tubes and plasma was isolated from the whole blood by centrifugation at 3,000 RPM for 5 minutes and stored at -80°C. Colonic tissue was harvested and fixed in either Histochoice^®^ Tissue Fixative (VWR) or methanol-based Carnoy’s fixative (60% absolute methanol, 30% chloroform, 10% glacial acetic acid), embedded in paraffin blocks, and 5 μm sections mounted on glass slides. Histochoice-fixed colon sections were stained with hematoxylin and eosin and evaluated for inflammation and pathology. Carnoy’s-fixed colon sections were stained with Periodic acid-Schiff to evaluate glycoprotein levels.

### Primary Intestinal Epithelial-Derived Spheroid Isolation and Culture

Crypts from 8 week old IL10KO mice were isolated and cultured as described (29). Briefly, whole colons were excised into Dulbecco’s Modified Eagle Medium (DMEM; Gibco) with penicillin/streptomycin, and 10% heat-inactivated fetal bovine serum (FBS); washing media. Colons were cut into 1cm long segments and shaken vigorously in ice cold PBS, then finely minced. Tissue was suspended in 2mL washing medium containing 2mg/mL collagenase type I powder (Gibco), and 50μg/mL gentamicin and incubated at 37°C for 30 minutes, with vigorous pipetting to further break apart the tissue into individual crypts. Samples were filtered through a 70μm cell strainer and washed a second time, at which point media was completely removed and crypts were suspended in growth factor reduced matrigel (Corning). Matrigel suspended crypts were added to center of 24 well tissue culture dish, and covered with 0.5mL of 50% L-WRN conditioned media (one part L-WRN conditioned media, one part Advanced DMEM-F12 with 2mM L-glutamine, 20% FBS, and penicillin/streptomycin) containing Y-27632 (Tocris) and A83-01 (Tocris). Media on spheroids was replaced on alternating days, with spheroids passaged every 3-4 days.

### Intestinal Epithelial Monolayer Culture

Colonic mouse spheroids were maintained for up to 30 passages in 50% L-WRN media containing Y-27632 and A83-01 for monolayer experiments as described (28). Matrigel encased spheroids were suspended in 0.5mL of PBS-EDTA and removed from plate, then incubated in trypsin. Washing medium was added and spheroids were pipetted vigorously fully dissociate spheroids into single cells and 2-3 cell aggregates. Approximately 30,000 cell aggregates on average in 0.2mL of media were seeded per apical chamber of a pre-coated 24 well transwell insert (Corning, 6.5mm diameter, 0.4μm pores). Transwell inserts were pre-coated for 20 minutes with 10% matrigel diluted in ice cold PBS. The basal chamber of each well contained 0.5mL of 50% L-WRN containing Y-27632 and A83-01. Medium was changed every other day. Medium in the apical chamber of the transwells was removed on day 7 to create an air-liquid interface (ALI). On day 25 of ALI culture, basal medium was replaced with experimental media. Experimental media consisted of one part L-WRN conditioned media and one part DMEM-F12 media without glucose, supplemented with 20% FBS (Life Sciences, Carlsbad, CA), 2mM L-glutamine, penicillin/streptomycin, and either D(+)-Glucose (4.5g/L; Fisher Scientific) or Maltrin (4.5g/L; Envigo). After 72 hours, monolayers were processed for histological or biochemical analysis. Monolayers were harvested for immunohistochemistry by fixing in a methanol-based Carnoy’s fixative (60% absolute methanol, 30% chloroform, 10% glacial acetic acid) for 24 hours. The transwell membrane and accompanying monolayer was cut in half and embedded upright in a 1% agar block. Agar blocks were embedded in paraffin, 5μm sections were mounted on glass slides, and the stained with Hematoxylin and Eosin (H&E) or Periodic Acid-Schiff (PAS).

### Enzyme-Linked Immunosorbent Assays

Serum Amyloid A levels were quantified in mouse plasma using the Mouse SAA ELISA kit (E-90SAA, Immunology Consultants Laboratory, Inc.) according to manufacturer’s specifications. Lipocalin-2 (LCN2) levels were assessed in mouse stool samples using R&D Systems Mouse Lipocalin-2/NGAL DuoSet ELISA (DY1857) and DuoSet Ancillary Reagent Kit 2 (DY008). Pre-weighed stool samples were suspended in 500μL of PBS and agitated overnight at 4°C by gentle rocking. Samples were vortexed at high speed until complete disruption of the stool pellet, and then centrifuged to pellet solid particulates. Supernatants were used to assay LCN2 levels following manufacturer’s specifications. Resulting levels of LCN2 in pg/mL were normalized to volume of PBS used for pellet suspension and dry weight of stool pellet to determine levels in ng/g of stool.

### Lamina Propria Immune Cell Isolation and Flow Cytometry

The proximal half of IL10KO colons were excised immediately after sacrifice, cut longitudinally to remove luminal contents, and sectioned into smaller pieces and placed on ice in 10mL of PBS/0.1% (w/v) bovine serum albumin (BSA) (Sigma) until further processing. To remove epithelial cells, PBS/0.1% BSA was aspirated and colon pieces were incubated in 15mL of PBS supplemented with EDTA (5mM final concentration) in an orbital shaker at 180 RPM for 15 minutes at 37°C. PBS/EDTA was aspirated and this step was repeated. Tissue was washed with PBS/0.1% BSA, buffer was removed and then the tissue was finely minced before being washed twice by incubation for 5 minutes at room temperature in 15 mL of complete media (RPMI 1640 with 3% (v/v) heat-inactivated fetal bovine serum (FBS), penicillin/streptomycin (100U/mL), and 20 mM HEPES). Complete media was removed and replaced with 10mL of digestion media (complete media containing 0.1mg/ml collagenase type VIII (Sigma, Cat. # C2139), and 0.075U/mL Dispase [BD Biosciences, Cat. # 354235)]. Samples were then incubated for 40 minutes at 37°C while shaking at 180 RPM. Tissue fragments were vortexed in digestion media, tissue allowed to gravity settle briefly (~1 minute), supernatants were harvested and strained through a 70µm cell strainer. 20mL of PBS/EDTA was passed through the cell strainer into the tube containing the cell digestion supernatant to maximize cell recovery and ensure that digestion was terminated. The remaining tissues were subjected to an identical second round of digestion, and the resulting cell-containing supernatants were pooled with those from the first digestion, and another 10mL of PBS/EDTA was used to wash the cell strainer and halt the digestion. Cells were pelleted by centrifugation at 513 x g for 10 minutes, the supernatant was discarded and the pellet was resuspended by flicking, and the volume remaining was measured and recorded for total cell enumeration. Cells were stored on ice until further processing. Cells were counted with a hemocytometer.

Cells were divided equally and placed in round-bottom 96-well plates for staining for flow cytometric phenotyping. For cytokine detection, cells were restimulated in RPMI 1640 with 5% (v/v) heat-inactivated FBS, penicillin/streptomycin (100U/mL) and 20 mM HEPES that was supplemented with 50ng/mL PMA (Sigma), 500ng/mL ionomycin (Sigma), and 1 x Brefeldin A (“Golgi Plug”; BD Bioscience) for 3 hours at 37°C. Cells that were not restimulated were used as controls for cytokine detection. Cells were washed twice to remove serum remaining from the digestion media (which interferes with fixable viability dyes), by addition of 200μL PBS/0.1% BSA, pelleting by centrifugation at 513 x g for 5 minutes, and discarding the supernatant. The pelleted cells were first incubated in PBS/0.1% BSA containing 50μg/mL anti-CD16/CD32 (clone 93; BioLegend) at 4°C for 20 minutes to prevent nonspecific binding of antibody in subsequent steps. Cells were stained with the following antibodies: CD45.2 Alexa 700 (104; BioLegend), TCR-β Alexa 488 (H57-597; BioLegend), TCRγδ phycoerythrin (PE)-Cy7 (GL3; BioLegend), IFN-γ peridinin chlorophyll protein (PerCP)-Cy5.5 (XMG1.2; BioLegend), IL-17A PE (TC11-18H10.1; BioLegend), RORγt allophycocyanin (APC) (B2D; eBio), FoxP3 eFluor-450 (FJK-16S; eBio), CD4 BV605 (RM4-5; BioLegend), CD11b PerCP-Cy5.5 or PE (M1/70; BioLegend), Ly6C PE or PerCP-Cy5.5 (HK1.4; BioLegend), Ly6G APC or PE-Cy7 (1A8; BioLegend), CD64 PE-Cy7 or APC (X54-5/7.1; BioLegend), MHCII Alexa 488 (M5/114.15.2; BioLegend), CD11c eFluor-450 (N418; Invitrogen), or appropriate fluorophore-conjugated isotype control antibodies purchased from the same supplier as the antibody used for the indicated targets. For exclusion of dead cells, the Zombie Aqua fixable viability kit was used (Biolegend). For surface staining, cells were resuspended in 50μL PBS/0.1% BSA containing a cocktail of fluorophore-conjugated antibodies. Cells were stored at 4°C in the dark for 20 minutes and were then washed twice with PBS/0.1% BSA as described above. For cells where no subsequent intracellular staining was performed, cell pellets were fixed by addition of 100μL of Fixation Buffer (BioLegend) and stored for 16 hours at 4°C before being washed twice with 200μL PBS/0.1%BSA and centrifugation at 912 x g. Cells were resuspended in 200μL PBS/0.1%BSA and acquired using an LSRFortessa flow cytometer (BD Biosciences). For cells where intracellular staining was performed cell pellets were fixed by addition of 100µL of Fixation/Permeabilization Buffer (eBioscience) (1 part concentrate to 3 parts diluent) and stored at 4°C for 16 hours. Cells were washed twice with 200μL of PBS/0.1%BSA as above, the supernatant was discard, cells were resuspended in 100μL permeabilization buffer (eBioscience) supplemented with normal rat serum (Sigma; 2% (v/v)), and placed in the dark for 30-60 minutes at 4°C. Cells were pelleted as above and resuspended in 50μL permeabilization buffer containing the appropriate cocktail of antibodies (anti- FoxP3, IFN-γ, IL-17A, RORγt, or isotype control) and stored in the dark at 4°C for 30 minutes. Cells were washed by addition of 200μL of permeabilization buffer and centrifuged at 912 x g for 5 minutes; the supernatant was discarded. This step was repeated twice, first with 200μL permeabilization buffer and subsequently with 200μL PBS/0.1% BSA. Cells were resuspended in 200μL PBS/0.1% BSA and acquired using an LSRFortessa flow cytometer (BD Biosciences). Data were further analyzed with FlowJo software (Tree Star).

### DNA Extraction and 16S rRNA Amplicon Sequencing

Cecal contents from both male and female IL10KO mice conditioned with NOD2KO microbiota and fed experimental diets from four independent experiments were collected for analysis. DNA was extracted as previously described (32). Briefly, DNA from cecal contents was isolated using the Qiagen QIAamp DNA Microbiome kit according to manufacturer’s specifications. Isolated microbial gDNA I was amplified at the V4 region of 16S rRNA using a nested PCR method and the addition of Illumina Nextera Unique Dual indexes. Libraries were pooled to ensure equal sample distribution amongst sequencing reads. Amplicon sequencing was performed on Illumina’s iSeq 100 with a 2 x 150 read length.

### Bioinformatics

Individual fastq files without non-biological nucleotides were processed using Divisive Amplicon Denoising Algorithm (DADA) pipeline (33). The output of the dada2 pipeline (feature table of amplicon sequence variants, ASV table) was processed for alpha and beta diversity analysis using *phyloseq*, and microbiomeSeq (http://www.github.com/umerijaz/microbiomeSeq) packages in R. Alpha diversity estimates were measured within group categories using estimate_richness function of the *phyloseq* package. Canonical Correspondence Analysis (CCA) was performed using Bray-Curtis dissimilarity matrix between groups and visualized by using the *ampvis2* package (34–36). Network analysis was performed using NetCoMi package. Multiple comparisons were adjusted for using the BH FDR method while performing multiple testing on taxa abundance across groups (37). Analysis of Variance (ANOVA) was used to assess alpha diversity. Permutational multivariate analysis of variance (PERMANOVA) was performed on all coordinates obtained during CCA using ord.res function in *phyloseq* package (38). Linear regression (parametric) and Wilcoxon (non-parametric) tests were performed on ASV abundances versus metadata variable levels (i.e. acetic acid levels) using R base functions.

### Short-Chain Fatty Acid Quantification

Cecal contents were hydrated with 0.005M NaOH, vortexed for 40 minutes at 4°C, and centrifuged to pellet solid particulates. A 30μL aliquot of supernatant was mixed with 50μL 2‐Butanol/Pyridine (3:2) containing the six heavy labeled internal standards. The carboxylic acids were then derivatized with isobutyl chloroformate. After derivatization, samples were mixed with hexane and the hexane layer was removed for GC‐MS analysis. The quantitation of acetic acid, butyric acid, and propionic acid was performed using isotope dilution GC‐MS/MS. The absolute quantity of each SCFA was determined using calibrations curves measured for each analyte. The abundance of each analyte in the samples was normalized to initial sample weight.

### Fluorescent *In Situ* Hybridization

Slides from Carnoy’s-fixed tissue samples were deparaffinized and hybridized with 500 ng Eub388-Alexa647 probe (or buffer-only controls) in 20mM Tris-HCL, 0.01% SDS, 0.9M NaCl at 50°C overnight. Slides were then rinsed twice with water, incubated 5 minutes in 20mM Tris-HCL, 0.9M NaCl at 46°C, rinsed twice with water, then dried 10 minutes at 46°C. Coverslips were mounted using Vectashield containing DAPI (Vector Laboratories Inc.) and visualized using an upright Leica SP5 multi-photon/confocal microscope (Leica). Image z-stacks were taken at 63x magnification and images collected every 0.45μm. A minimum of 4 fields per sample were analyzed, with 2 samples per diet group from three independent experiments. The distance between the microbiota and the epithelium was measured using ImageJ (National Institutes of Health) image analysis software. For each field, 5 measurements were averaged together.

### Immunofluorescent Staining and Microscopy

Slides from Carnoy’s-fixed tissue samples were deparaffinized and subjected to antigen retrieval by steaming for 30 minutes in a sodium citrate buffer (10mM sodium citrate, pH 6.0, 0.05% Tween 20). Sections were blocked (HBSS, 2% goat serum, 2% FBS, 0.4% Triton X-100) and stained with antibodies against Muc2 (NBP1-31231; Novus Biologicals; diluted 1:500), Ki67 (ab15580; Abcam; diluted 1:100), or E-cadherin (CM1681; ECM Biosciences; diluted 1:200). Sections were then incubated with goat anti-rabbit Alexa Fluor 568 (Invitrogen) or goat anti-mouse Alexa Fluor 488 (Invitrogen) diluted 1:1,000-2,000. Coverslips were mounted using Vectashield containing DAPI (Vector Laboratories Inc.) and visualized using an upright Leica SP5 multi-photon/confocal microscope (Leica) or wide field microscope where indicated. For confocal microscopy, image z-stacks were taken at 40x magnification and images collected every 0.45μm; wide field images were taken at 20x magnification. Approximately 3-5 fields per sample were analyzed, with 2 samples per diet group from three independent experiments. The number of Ki67-positive nuclei present in the epithelial layer were counted in each field, the normalized to the number of crypts present in the section.

### Mucus Quantification

PAS stained slides were scanned into electronic files using an Aperio AT2 slide scanner at 20x magnification. Aperio ImageScope software was used to manually count PAS-positive (PAS+) cells per tissue section. Area of tissue samples was determined using ImageJ (National Institutes of Health) image analysis software. Secreted mucus area was quantified using ImageJ image analysis software.

### Intestinal Epithelial Cell Isolation

The proximal half of IL10KO colons were excised immediately after sacrifice, cut longitudinally to remove luminal contents, and placed in HBSS on ice. Tissue samples were removed from the buffer and finely chopped, then transferred to a tube with 15mL of pre-warmed Buffer B (HBSS, 2.5% penicillin/streptomycin, 0.625mM HEPES, 0.05mM EDTA, 90μM DTT). Samples were incubated at 37°C for fifteen minutes, and vortexed for 30 seconds every 5 minutes. Samples were then centrifuged at 500 RPM for 2 minutes. Supernatants were collected in a new tube, 15mL of Buffer B was used to resuspend the cell pellet, and the incubation and supernatant collection process was repeated for a total of three times. The collected supernatants were centrifuged at 1,500 RPM for 5 minutes, cell pellets washed with 5mL of PBS, and then resuspended in 1mL of PBS. Suspended cells were divided equally into two 1.5mL Eppendorf tubes, centrifuged at 9,000 RPM for 5 minutes at 4°C, and then suspended in either 1mL of Trizol (ThermoFisher Scientific) or 250μL RIPA buffer (150mM NaCl, 1% NP-40, 50mM Tris, pH8, 0.5% sodium deoxycholate, 0.1% SDS).

### RNA Transcript Analysis

RNA was extracted from epithelial monolayers using the RNeasy Mini Kit (Qiagen) according to manufacturer’s specifications. Isolated intestinal epithelial cells were homogenized in TRIzol (ThermoFisher Scientific) and RNA extracted according to manufacturer’s specifications. Samples were then DNase1 treated and cleaned using the RNeasy Mini Kit (Qiagen) according to manufacturer’s specifications. RNA was converted to cDNA using the BioRad iScript cDNA synthesis kit, and qPCR performed in duplicate using SYBR Green supermix. Values were quantified using the 2^-ΔΔCT^ method (39). Primer sequences are listed in **Supplementary Table 2**.

### Statistical Analyses

Statistical analyses was performed using GraphPad Prisms software (version 9.1.0). For data sets with multiple variables, one-way or two-way ANOVA was used to determine significance with *post-hoc* Dunnett’s multiple comparison test. For parametric data sets with only two variables, unpaired, two-tailed, Student’s t-test was used to determine significance. For non-parametric data sets with only two variables, unpaired, two-tailed, Mann-Whitney test was used to determined significance.

## Data Availability Statement

The datasets presented in this study can be found in online repositories. The names of the repository/repositories and accession number(s) can be found below: European Nucleotide Archive, accession no: PRJEB49815.

## Ethics Statement

The animal study was reviewed and approved by Cleveland Clinic Institutional Animal Care and Use Committee.

## Author Contributions

MZ and CM conceived and supervised the study. MZ, AP, NM, ME, PA, and NS performed experiments and analyzed data. MZ and CM wrote the manuscript. All authors contributed to the article and approved the submitted version.

## Funding

This work was supported by funds from Cure4IBD (to CM), and the McDonald Family Trust (to CM). Opinions, interpretations, conclusions, and recommendations are those of the authors and are not necessarily endorsed by the funders.

## Conflict of Interest

The authors declare that the research was conducted in the absence of any commercial or financial relationships that could be construed as a potential conflict of interest.

## Publisher’s Note

All claims expressed in this article are solely those of the authors and do not necessarily represent those of their affiliated organizations, or those of the publisher, the editors and the reviewers. Any product that may be evaluated in this article, or claim that may be made by its manufacturer, is not guaranteed or endorsed by the publisher.
