# Supplementary material for: Maltodextrin Consumption Impairs the Intestinal Mucus Barrier and Accelerates Colitis Through Direct Actions on the Epithelium
Source: Front Immunol. 2022 Mar 14;13:841188. doi: 10.3389/fimmu.2022.841188 (PMC8963984; doi:10.3389/fimmu.2022.841188)
Supplement: Supplementary file 1 [file DataSheet_1.docx]

Supplementary Material

**Supplementary Figure 1.** (**A**) Colitis score broken down into individual sub-scores. Each sub-score has a maximum score of 4. Mean±SEM graphed, *p<0.05, ***p<0.001 by 2-way ANOVA with Dunnett’s multiple comparisons test. Submuscosal swelling sub-scores also trended higher in the food additive fed groups, but did not achieve statistical significance (p=0.074, MDX; p=0.054, CMC). (**B**) Disease activity index score at endpoint. The score is a cumulative total of the 5 assessed parameters (weight loss, stool consistency, posture, grooming, and rectal prolapse), with a max score of 10. (**C**) Weight change as a percent of the weight in grams at the start of the experiment. (**D**) Weight of food consumed weekly averaged per mouse. Food was weighed weekly, and starting weight subtracted from ending weight to determine amount consumed. Total consumed food weight was normalized by the number of mice present in the cage.

**
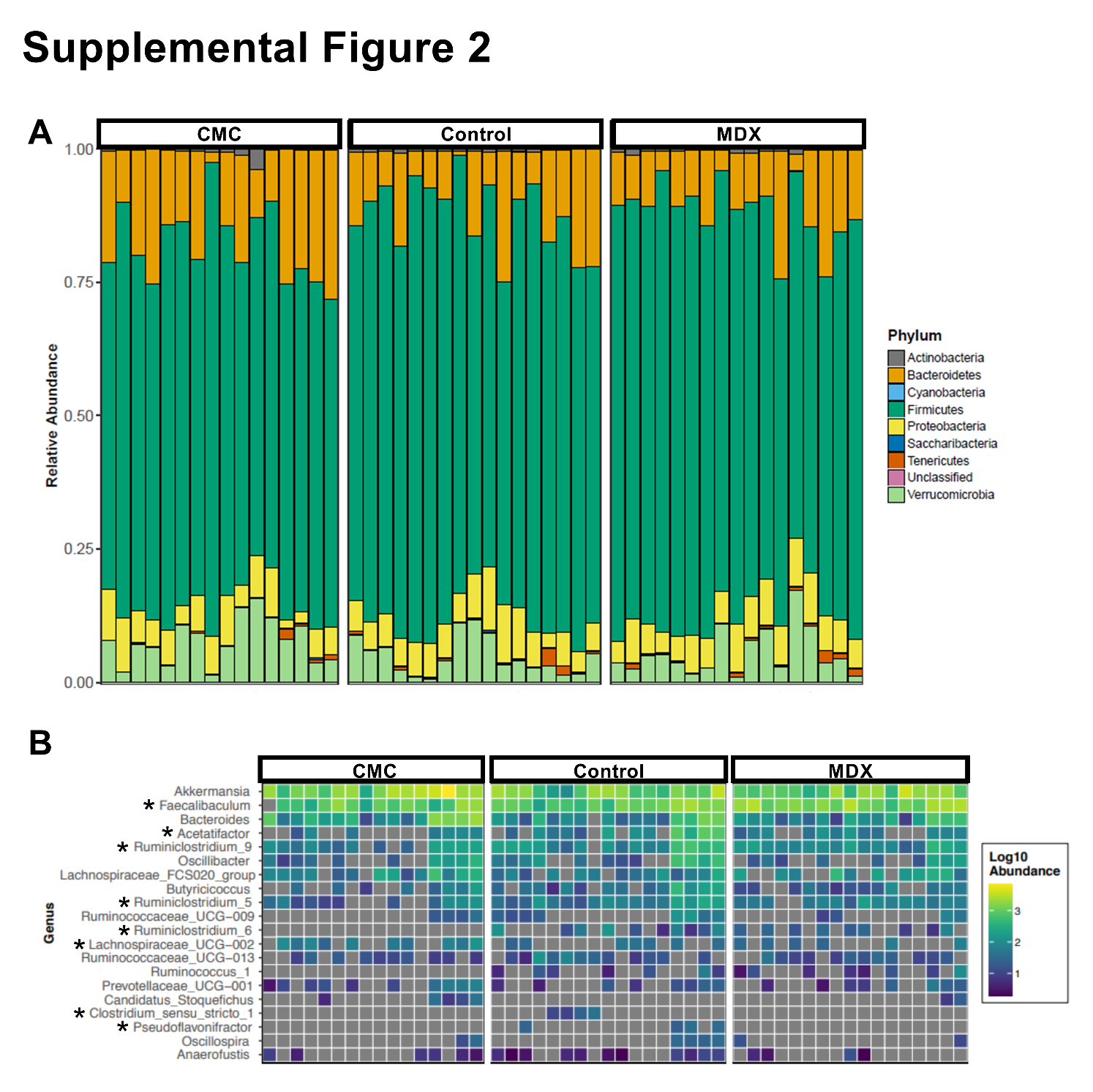
**

**Supplementary Figure 2.** (**A**) Relative abundance of 9 bacterial phyla as assessed by 16S rRNA sequencing of cecal contents from IL10KO mice. (**B**) Heat map displaying the altered abundance of 20 genera. Statistically significant alterations in abundance are marked with an asterisk.

**
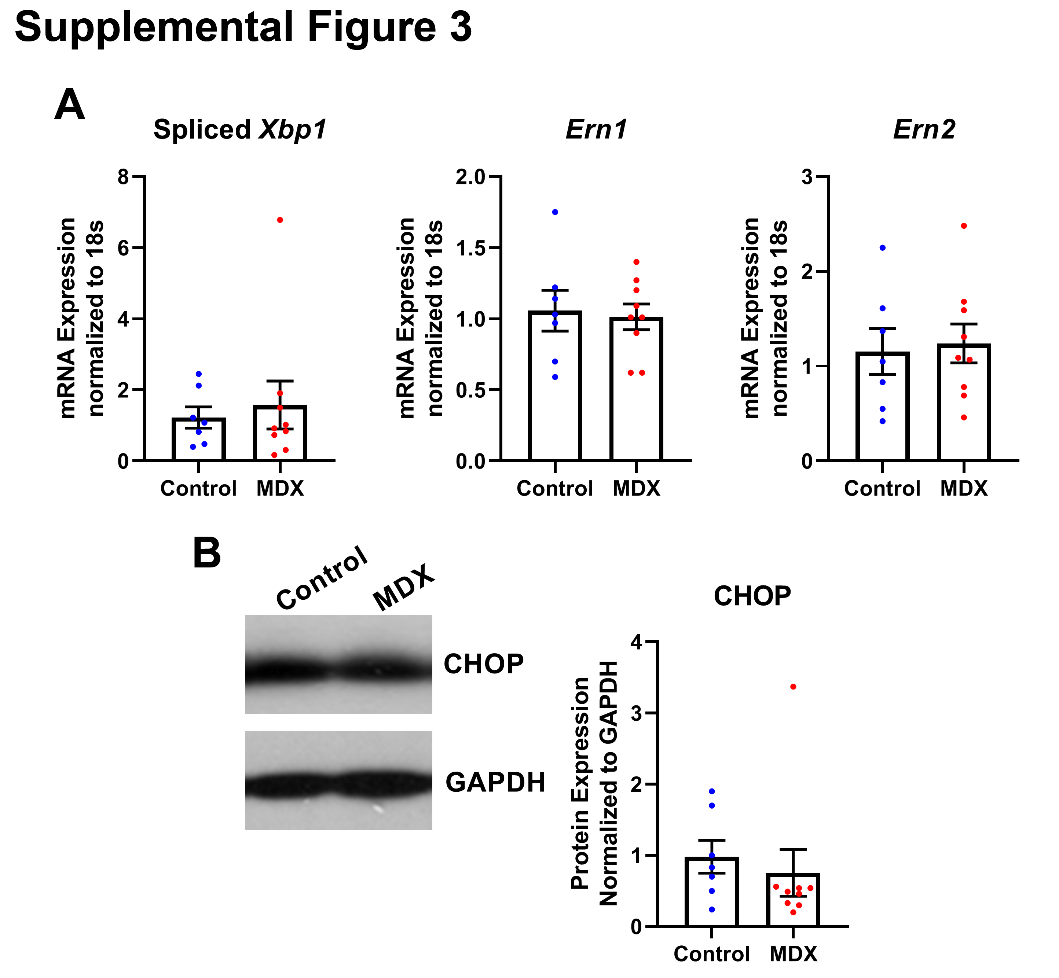
**

**Supplementary Figure 3.** (**A**) Assessment of ER stress pathway signaling components in whole colon tissue from IL10KO mice fed a control or MDX-supplemented diet by qRT-PCR. Relative expression level of spliced *Xbp1, Ern1,* and *Ern2* assessed by qRT-PCR. All expression levels normalized to 18S ribosomal transcript levels (n=7-9 mice/group, mean±SEM). (**B**) Protein levels of CHOP were assessed by immunoblot and quantified by densitometric analysis relative to GAPDH levels (n=7-9 mice/group, mean±SEM).

## Supplementary Table 1. Genes and associated primer sequences used for qRT-PCR analysis.

| **Target Gene** | **Forward primer** | **Reverse primer** |
| --- | --- | --- |
| *Bmi1* | AAACCAGACCACTCCTGAACA | TCTTCTTCTCTTCATCTCATTTTTGA |
| *Hopx* | AGACGCAGAAATGGTTTAAGC | TCCAAGAGCAAGCTCAAGGG |
| *Klf4* | GATTAAGCAAGAGGCGGT | GGTAAGGTTTCTCGCCTGTG |
| *Lgr5* | CATCTAGGCGCAGGGATTGA | GTCTCCTACATCGCCTCTGC |
| *Math1* | AAAGGAGGCTGGCAGCAA | TGGTTCAGCCCGTGCAT |
| *Spdef1* | GGAGAAGGCAGCATCAGGA | CCAGGGTCTGCTGTGATGT |
| 18s | CATTCGAACGTCTGCCCTAT | CCTGCTGCCTTCCTTGGA |

## Supplementary Table 2. Full taxa names from microbial network analysis.

| ASV | Phylum | Class | Order | Family | Genus |
| --- | --- | --- | --- | --- | --- |
| 1 | Firmicutes | Erysipelotrichia | Erysipelotrichales | Erysipelotrichaceae | Allobaculum |
| 10 | Proteobacteria | Deltaproteobacteria | Desulfovibrionales | Desulfovibrionaceae | Desulfovibrio |
| 11 | Firmicutes | Bacilli | Lactobacillales | Lactobacillaceae | Lactobacillus |
| 16 | Verrucomicrobia | Verrucomicrobiae | Verrucomicrobiales | Verrucomicrobiaceae | Akkermansia |
| 24 | Firmicutes | Clostridia | Clostridiales | Lachnospiraceae | NA |
| 59 | Firmicutes | Clostridia | Clostridiales | Lachnospiraceae | Lachnospiraceae_  FE2018_group |
| 62 | Firmicutes | Clostridia | Clostridiales | Lachnospiraceae | Lachnospiraceae_UCG-006 |
| 66 | Bacteroidetes | Bacteroidia | Bacteroidales | Bacteroidales_S24-7 | NA |
| 100 | Firmicutes | Clostridia | Clostridiales | Lachnospiraceae | NA |
| 101 | Firmicutes | Erysipelotrichia | Erysipelotrichales | Erysipelotrichaceae | Turicibacter |
| 102 | Bacteroidetes | Bacteroidia | Bacteroidales | Bacteroidales_S24-7 | NA |
| 108 | Firmicutes | Clostridia | Clostridiales | Lachnospiraceae | NA |
| 113 | Firmicutes | Erysipelotrichia | Erysipelotrichales | Erysipelotrichaceae | Turicibacter |
| 147 | Firmicutes | Clostridia | Clostridiales | Lachnospiraceae | Lachnospiraceae_NK4A136_group |
| 149 | Firmicutes | Clostridia | Clostridiales | Lachnospiraceae | Lachnospiraceae_NK4A136_group |
| 171 | Firmicutes | Clostridia | Clostridiales | Lachnospiraceae | Lachnospiraceae_NK4A136_group |
| 186 | Bacteroidetes | Bacteroidia | Bacteroidales | Bacteroidales_S24-7 | NA |
| 208 | Firmicutes | Clostridia | Clostridiales | Ruminococcaceae | Ruminococcaceae_UCG-014 |
| 261 | Firmicutes | Clostridia | Clostridiales | Peptococcaceae | NA |
| 285 | Firmicutes | Clostridia | Clostridiales | Lachnospiraceae | NA |
| 320 | Firmicutes | Erysipelotrichia | Erysipelotrichales | Erysipelotrichaceae | Erysipelatoclostridium |
| 321 | Firmicutes | Bacilli | Lactobacillales | Lactobacillaceae | Lactobacillus |
| 323 | Firmicutes | Clostridia | Clostridiales | Lachnospiraceae | Acetatifactor |
| 332 | Actinobacteria | Coriobacteriia | Coriobacteriales | Coriobacteriaceae | Senegalimassilia |
| 335 | Firmicutes | Clostridia | Clostridiales | Ruminococcaceae | Intestinimonas |
| 336 | Bacteroidetes | Bacteroidia | Bacteroidales | Bacteroidales_S24-7 | NA |
| 353 | Bacteroidetes | Bacteroidia | Bacteroidales | Bacteroidales_S24-7 | NA |
| 355 | Firmicutes | Clostridia | Clostridiales | Lachnospiraceae | NA |
| 361 | Firmicutes | Erysipelotrichia | Erysipelotrichales | Erysipelotrichaceae | Erysipelatoclostridium |
| 375 | Firmicutes | Bacilli | Lactobacillales | Enterococcaceae | Enterococcus |
| 409 | Bacteroidetes | Bacteroidia | Bacteroidales | Bacteroidales_S24-7 | NA |
| 412 | Firmicutes | Clostridia | Clostridiales | Lachnospiraceae | NA |
| 468 | Firmicutes | Clostridia | Clostridiales | Lachnospiraceae | Lachnospiraceae_UCG-002 |
| 470 | Firmicutes | Clostridia | Clostridiales | Lachnospiraceae | Roseburia |
| 510 | Firmicutes | Clostridia | Clostridiales | Lachnospiraceae | NA |
| 514 | Proteobacteria | Gammaproteobacteria | Enterobacteriales | Enterobacteriaceae | NA |
| 546 | Firmicutes | Clostridia | Clostridiales | Lachnospiraceae | NA |
| 571 | Bacteroidetes | Bacteroidia | Bacteroidales | Prevotellaceae | Prevotellaceae_  UCG-001 |
| 576 | Firmicutes | Clostridia | Clostridiales | Ruminococcaceae | NA |
| 590 | Bacteroidetes | Bacteroidia | Bacteroidales | Bacteroidaceae | Bacteroides |
| 593 | Firmicutes | Clostridia | Clostridiales | Lachnospiraceae | NA |
| 598 | Firmicutes | Clostridia | Clostridiales | Ruminococcaceae | Oscillibacter |
| 624 | Firmicutes | Clostridia | Clostridiales | Lachnospiraceae | NA |
| 662 | Firmicutes | Clostridia | Clostridiales | Lachnospiraceae | NA |
| 749 | Bacteroidetes | Bacteroidia | Bacteroidales | Bacteroidales_S24-7 | NA |
| 856 | Bacteroidetes | Bacteroidia | Bacteroidales | Rikenellaceae | NA |
| 860 | Firmicutes | Clostridia | Clostridiales | Ruminococcaceae | Acetanaerobacterium |
| 901 | Firmicutes | Clostridia | Clostridiales | Clostridiales_  vadinBB60 | NA |
